# Sexual Consent Norms in a Sexually Diverse Sample

**DOI:** 10.1007/s10508-023-02741-0

**Published:** 2023-11-28

**Authors:** Emily A. Harris, Thekla Morgenroth, Damien L. Crone, Lena Morgenroth, Isabel Gee, Harry Pan

**Affiliations:** 1 Melbourne School of Psychological Sciences, Faculty of Medicine, Dentistry and Health Sciences, University of Melbourne, Parkville, VIC 3010 Australia; 2 Department of Psychological Sciences, Purdue University, West Lafayette, IN USA; 3 Positive Psychology Center, University of Pennsylvania, Philadelphia, PA USA; 4 Berufsverband erotische und sexuelle Dienstleistungen e.V., Berlin, Germany; 5 Department of Psychology, University of Exeter, Exeter, UK; 6 School of Psychology, University of Queensland, Brisbane, QLD Australia

**Keywords:** BDSM, Natural language processing, Norms, Sexual consent, Sexual minority

## Abstract

Sexual consent has received increased attention in mainstream media, educational, and political settings since the rise of the #MeToo movement in 2017. However, long before #MeToo, sexual consent has been a core practice among people who engage in Bondage and Discipline, Dominance and Submission, Sadism, and Masochism (BDSM). This study examined sexual consent norms among a sexually diverse sample, including people who practice BDSM (*n* = 116), people who identify with another sexual minority group, such as swingers and sex workers (*n* = 114), and people who did not identify with a sexual minority group, termed sexual majority group members (*n* = 158). Explicit consent for both BDSM- and non-BDSM-related activities was rated as more common (descriptively normative) among people who were a member of the BDSM community compared to majority participants. Further, BDSM participants rated consent discussions as less sexually disruptive compared to majority participants. We found no significant group differences in the extent to which people thought sexual consent should be discussed. We also discuss findings from an open-ended question asking participants to recall a recent sexual experience with a new partner. This study demonstrates variability in consent norms between groups and points to the potential to shift sexual consent behaviors among majority participants.

**Supplementary Information:**

The online version contains supplementary material available at 10.1007/s10508-023-02741-0.

## Introduction

Many people do not receive adequate sex education in school (Flores & Barroso, 2017; MacDougall et al., 2020; Widman et al., 2016; Willis et al., 2019), and people tend not to discuss the details of their sexual experiences publicly (Afifi et al., 2008; Mackay, 2000; Widman et al., 2016). So, how do people develop an idea of what is normative sexual behavior? They may rely on alternative sources of information, such as film, television, porn, and other media sources to develop an understanding of what is considered “normal” when it comes to sex (von Rosen et al., 2017). They may also rely on discussions with select friends and parents (Bleakley et al., 2009; Fisher et al., 2019). A known gap in formal and informal sex education is sexual consent (Cary et al., 2022; Padilla-Walker et al., 2020; Widman et al., 2016; Willis et al., 2019). The lack of education about sexual consent is reflected in low rates of explicit sexual consent discussions (Muehlenhard et al., 2016). Below, we define sexual consent and detail why it is an important, though rarely practiced, part of sexual activity. We then discuss sexual norms, sexual consent norms, and consent among different sexual communities. We focus predominantly on research conducted with Western samples.

### What Is Sexual Consent?

Muehlenhard et al. (2016) presented three definitions of “sexual consent” that have been used in the literature and in practice. First, sexual consent can be defined as an internal state of willingness to engage in sexual behavior, where, “sexual” is used broadly to refer to behaviors people perceive to be erotic. Muehlenhard et al. noted, however, that an internal state of willingness to have sex is unknowable by others, and, therefore, is practically unhelpful for defining sexual consent. A second conceptualization of consent is a behavior others may interpret as willingness to have sex. Consent behaviors could be active (e.g., kissing the other person) or passive (e.g., not resisting being undressed). This is referred to as “inferred” consent. Muehlenhard et al. (2016) note that one flaw of inferred consent is that behaviors can be ambiguous. For example, not resisting a sexual action may or may not indicate willing consent. Thus, inferred consent is also unhelpful for defining consent because it focuses on another person’s interpretation of a behavior, rather than a person’s intention to consent.

Lastly, consent can be defined as an explicit agreement to engage in sexual behavior. Explicit agreements can include verbal statements like “Can I kiss you?” followed by a verbal “yes” or a nod. Explicit consent overlaps with “affirmative consent,” which has been defined as “affirmative, conscious, and voluntary agreement to engage in sexual activity…Lack of protest or resistance does not mean consent, nor does silence mean consent” (Office of the Press Secretary, 2014).

Explicit consent distinguishes between wanting and consenting to something (Muehlenhard et al., 2016). For example, a couple may not want to have sex but consent because they are trying to have a baby. Similarly, someone may want to have sex with someone but not consent because they are in a monogamous relationship with someone else. In such situations, an internal willingness or non-verbal cue of arousal (or the lack thereof) would be misleading and would not constitute consent. While explicit consent is the least ambiguous, and many people formally define sexual consent in this way, in practice, it is the least commonly used form of consent (Muehlenhard et al., 2016). People rely predominantly on inferred consent (Curtis & Burnett, 2017; Graf & Johnson, 2021; for review, see Muehlenhard et al., 2016).

### Why Is Sexual Consent Important?

Scholars have noted that the topic of sexual consent is both simple and complicated (Harris, 2018). Sexual consent is simple in that if a person does not consent to sexual activity, then it is a case of sexual misconduct, abuse, harassment, and/or rape. Additionally, the high rates of non-consensual sex around the world represent a major social problem. The scope of this problem was highlighted by the rise of the #MeToo movement (Carlsen et al., 2018; Zacharek et al., 2017), started by Tarana Burke in 2006 to raise awareness of sexual abuse and build a community of support for victims (Garcia, 2017; Suk et al., 2021). Since the popularization of the #MeToo movement in 2017, sexual consent education has been centered in efforts to redress sexual misconduct (Cary et al., 2022; Harris, 2018; Jaffe et al., 2021; O’Neil et al., 2018).

Sexual consent is, however, complicated in part because explicit consent is rarely practiced due to deeply ingrained (hetero) sexual scripts. The dominant cultural narrative that dictates what sex looks like, and should look like, is referred to as the “traditional sexual script” (Buhi et al., 2010; Jozkowski et al., 2019; Sakaluk et al., 2014). The traditional sexual script assumes that men should initiate sexual behavior and women, as the sexual gatekeepers, should verbally signal a lack of consent (Byers, 1996; O’Sullivan & Byers, 1992; Simon & Gagnon, 1984). If she does not, sexual consent is assumed (Wiederman, 2005). It is also normative for women to feign resistance in line with gendered assumptions about women’s sexual purity (Muehlenhard et al., 2016; O’Sullivan & Byers, 1992). Thus, within the traditional sexual script, consent is inferred unless stated otherwise, and if explicit non-consent is stated, it is framed as ambiguous.

Of course, individuals can and do diverge from the traditional sexual script (Dworkin & O’Sullivan, 2005; Masters et al., 2013; Morrison et al., 2015)—for example, many women initiate sex and many men prefer egalitarian or women-led sexual initiation (Dworkin & O’Sullivan, 2005). Sexual scripts nonetheless set a cultural understanding of what can be expected during sex that impacts sexual behavior. Consistent with the narrative that people engaged in sex are driven by “uninterrupted passion” (Gagnon, 1990; Masters et al., 2013; O’Sullivan & Byers, 1992), people tend to perceive verbal discussion of sexual consent as disruptive, stifling (Curtis & Burnett, 2017; Humphreys & Herold, 2003), (Beres, 2010), “awkward,” and “weird” (Curtis & Burnett, 2017). Thus, while sexual consent is clearly important, there are societal-level barriers to engaging in explicit consent discussions. Namely, sexual consent is perceived as non-normative and disruptive.

Sexual consent is also complicated in part because it should not be considered a panacea for sexual misconduct (Gavey, 2018; Harris, 2018). Sexual misconduct can and does occur when sexual non-consent (i.e., “no”) is explicitly communicated and understood. In such cases, sexual consent communication is not the problem, but rather, broader ideological and personal factors, such as misogyny and sexual entitlement (Abbey et al., 2011; Gavey, 2018; MacKinnon, 2018). Further, Harris (2018) notes the importance of understanding consent beyond “yes” and “no.” A simple “yes” and “no” may not be sufficient for sexual consent or non-consent and should be understood within personal and systemic contexts, such as interpersonal power relations. For example, a person may explicitly consent to sex due to social and economic pressures, such as if a boss or supervisor makes a sexual advance.

Taking these points together, sexual consent is a key component of ethical sex, and education about consent should extend beyond “yes” and “no.” While sexual consent literacy will not solve the issue of sexual misconduct, critical engagement with sexual consent may lead to positive changes to the traditional sexual script. Centering sexual consent may help to foreground mutual pleasure and the relational nature of sex. Such a shift can have implications at the individual and societal levels, including potential reductions in instances of unpleasurable, unwanted, and non-consensual sex.

One avenue for updating the traditional sexual script is considering existing, consent-focused sexual scripts. Sexual scripts describe a sequence of activities. The traditional sexual script includes activities that are perceived to be normative, such as kissing, oral sex, and penetrative sex. As noted above, explicit sexual consent discussions tend to be perceived as non-normative and are, therefore, not incorporated into the traditional sexual script. Next, we briefly review the literature on norms and the effects of group membership on sexual consent norms.

### Sexual Consent Norms and Group Membership

Social norms represent heuristics that people use to guide their attitudes and behaviors, including sexual attitudes and behaviors (Cialdini et al., 1991). The literature distinguishes between descriptive and injunctive norms (Cialdini et al., 1991; Jacobson et al., 2011). Descriptive norms refer to perceptions of how people in your social circle tend to think and act. For example, if you see friends purchase condoms, there is a descriptive norm to have and use condoms. Injunctive norms refer to how people in your social circle believe others ought to think and act. For example, if your friends purchase condoms and say, “you should make sure you’ve got condoms with you,” then there is also an injunctive norm of buying condoms (Cialdini et al., 1991; Jacobson et al., 2011).

When it comes to sexual consent, it can be difficult to assume what is descriptively or injunctively normative since people’s social circles may not openly discuss sexual consent (Widman et al., 2016), and formal and informal sex education often exclude sexual consent topics (e.g., Cary et al., 2022; Willis et al., 2019). Moreover, explicit consent is considered disruptive and stifling (Curtis & Burnett, 2017; Humphreys & Herold, 2003). In the absence of clear community norms, people may be likely to revert to following a traditional sexual script, which does not incorporate explicit discussions of consent (Cary et al., 2022; Jozkowski & Peterson, 2013).

However, there may be group differences in the extent to which media influence consent norms. Popular media tends to depict heterosexual sex and may be less likely to inform the sexual scripts of people who do not engage in heterosexual sex (Bauer, 2021; de Heer et al., 2021; Sternin et al., 2022). For example, Sternin et al. (2022) found that explicit consent was perceived to be more common among non-heterosexual men than heterosexual men, in part because they cannot rely on (heteronormative) sexual scripts to navigate sexual encounters. Further, there may be group differences in the likelihood of openly discussing sexual experiences and consent.

One community in which explicit discussions of sexual consent may be particularly normative is the Bondage and Discipline, Dominance and Submission, Sadism and Masochism (BDSM) community (Pitagora, 2013; Zambelli, 2017). BDSM interactions often occur in a specific setting and time called a “scene,” in which a range of activities, such as domination, humiliation, and the administration of pain, are carried out (Sagarin et al., 2009). Often, these activities are discussed and agreed upon in advance. In addition, BDSM interactions often include the use of safe words, which indicate the desire to stop the current BDSM interaction and override any power dynamic of the BDSM situation. Moreover, verbal processing after a scene to confirm that the experience was consensual is common. Sexual consent discussions are critical and necessary for distinguishing between BDSM activities and abuse (Dunkley & Brotto, 2020; Taylor & Ussher, 2001).

Consent emerged as one of the key themes in Taylor and Ussher's (2001) study, in which Taylor and Ussher interviewed 24 self-identified sadomasochists. For example, one participant noted: “SM is about consent...if there’s no consent it’s not SM...it’s sexual violence...it’s as simple as that” (p. 297). Carlström (2017) reported similar results in an interview study of Swedish BDSM community members. It makes sense that BDSM practitioners apply explicit rules of consent as they cannot use expressions of pain or discomfort to infer consent—or the lack thereof—and where the intentional testing of boundaries is one of the central aspects of many BDSM encounters (Taylor & Ussher, 2001). Members of the BDSM community have developed clear community guidelines and manuals describing how consent should be discussed (Dunkley & Brotto, 2020). The BDSM community appears to have clear descriptive consent norms (e.g., prescene negotiation, safe words, and post-scene discussion) and injunctive consent norms (e.g., community guidelines), whereby explicit verbal consent is critical before, during, and after BDSM activity. Importantly, some researchers have noted that while consent is understood to be central and necessary for any BDSM practices, there are cases of consent violations (Fanghanel, 2020). Fanghanel (2020) notes the continued importance of highlighting consent as central to BDSM practice.

Once sexual consent norms are ingrained, they may not be restricted to BDSM contexts. Instead, members of the BDSM community may find explicit discussions of sexual consent more normative and less disruptive and apply similar principles to non-BDSM interactions. This generalization of consent norms is in line with Barker's (2013) observation that members of the BDSM community have argued that explicit discussions of consent should be adopted more widely, even in non-sexual situations (e.g., asking someone if they would like an alcoholic drink, and relatedly, respecting others’ decision not to drink).

The idea that norms will influence behavior is consistent with social identity theory, which posits that the groups we belong to shape our attitudes and behaviors (e.g., Ellemers & Haslam, 2012). The extent to which we believe that our group (e.g., BDSM community members) engages in explicit sexual consent discussions may shape the likelihood that we have sexual consent discussions. To our knowledge, there has been no quantitative test of whether consent discussions are more normative or common among members of the BDSM community compared to non-members.

### The Current Study

This study investigates explicit consent norms and behaviors in different sexual communities. We aim to conduct the first quantitative study of consent norms, perceptions of disruptiveness, and the likelihood of discussing consent among people who do and do not practice BDSM. We aim to expand on previous research by focusing on consent norms for BDSM behaviors and non-BDSM sexual behaviors.

In line with the literature described above, we predict the following:

#### H1

Compared to people who don’t belong to the BDSM community, members of the BDSM community will be more likely to view explicit discussions of consent for non-BDSM behaviors as:

- More common in their social circles (descriptively normative)
- More imperative in their social circles (injunctively normative), and,
- Less disruptive

#### H2

Compared to people who don’t belong to the BDSM community, members of the BDSM community will be more likely to describe an explicit consent discussion when recounting a recent sexual experience with a new partner than those who don’t belong to the BDSM community.

Our study focuses primarily on the BDSM community, since sexual consent is central to their sexual scripts. In addition, we assess a secondary hypothesis that members of other sexual minority communities may be more likely to discuss consent and perceive consent as normative and non-disruptive. Members of other sexual minority groups may be less likely to adopt the traditional heterosexual script and hence may be more likely to incorporate active discussion and negotiation with new partners (e.g., Bauer, 2021; Beres et al., 2004).^1^ However, sexual consent may be less normative among other sexual minority group members than BDSM community members if sexual consent is not as central to their sexual script.

## Method

### Participants

We recruited participants via various online channels. First, we listed our study on several websites related to sex and relationship psychology, namely http://www.lehmiller.com, http://www.scienceofrelationships.com, and http://www.socialpsychology.org. Moreover, as we were interested in recruiting participants from different sexual communities, particularly the BDSM community, we posted our study on different subreddits (forums) on reddit.com (e.g., r/sex, r/BDSM, r/BDSMcommunity).

Our final sample consisted of 388 participants with an average age of 31.35 years (SD = 13.35). Half of our participants (49%) identified as women, while 40% identified as men and 2% as non-binary. The remaining 8% of participants did not indicate their gender. Almost half of our participants (46%) came from the USA, followed by 9% from the UK, 5% from Canada, 4% from Germany, and 3% from Australia. The remaining participants selected “other” (11%) or did not indicate their nationality (22%). Our sample was overall highly educated, with 18% indicating that their highest degree was a post-graduate degree such as MSc or Ph.D., 34% indicating they obtained an undergraduate degree, and 34% indicating that they had attended at least some university classes. Ten percent of participants indicated that their highest educational qualification was their high school (or equivalent) degree, and only 1.5% reported that they had not graduated from high school. Most participants (60.8%) were in one or more committed relationships. The sample overall indicated that they were fairly sexually experienced (*M* = 5.13; *SD* = 1.46; on a 1–7 scale) and had an average of 25.90 (*SD* = 121.48) sexual partners and a median of 10. The number of sexual partners ranged from 0 to 2000, with all responses over 150 belonging to sex workers.

Of those who indicated how they had found the survey, 47.4% came from Reddit, followed by 14.1% who participated as part of a class or class assignment (advertised via link sharing, not by the research team), and 11.1% had found the survey through http://www.lehmiller.com. All other sources, such as Google searches, word of mouth, and other websites, made up less than 10%.

We successfully recruited a sexually diverse sample. A total of 31% of our sample identified as members of the LGBTIQ+ community (1.5% lesbian, 3.1% gay, 22.2% bisexual, 7.2% pansexual, 2.1% asexual, 1.5% trans*, 0% intersex, 4.6% queer, 3.4% genderqueer, 3.9% questioning, 2.3% “other”). Thirty percent of participants were members of the BDSM community, and an additional 23% were members of another minority community but not the BDSM community (4.1% crossdressing, 15.2% swinger, 14.4% polyamory, 3.9% sex work, and 6.4% tantra). Participants could select multiple options, so these groups are not mutually exclusive.

### Procedure

We advertised the study as a survey of sexual attitudes and behaviors. After clicking a link, participants were informed about the study and asked to consent. We asked participants their ages, and the survey ended for participants younger than 18. We asked participants to first describe their most recent sexual event with a new partner, followed by measures of descriptive and injunctive norms, perceptions of consent as disruptive, sexual background questions, and demographic questions.

### Measures

Below, we briefly outline our key measures. We include detailed item descriptions in the online supplementary material.

#### Sexual Community

We asked participants whether they were members of the following communities: BDSM, LGBTIQ+ , fetish, crossdressing, swinger, poly, sex work, and tantra, with the option of indicating additional community memberships. Participants could select “yes” for multiple communities.

#### Descriptive and Injunctive Norms

We assessed descriptive norms related to discussions of sexual consent using the question stem “Most people in my social circles…”, followed by nine statements about non-BDSM sexual activities (e.g., “…would ask for permission before stimulating a new partner anally” *α* = 0.80) and eight statements about BDSM activities (e.g., “…would verbally ask for permission before spanking a new partner” *α* = 0.84). We instructed participants to “focus on how people would act, even if they don’t actually engage in these behaviors.” See the online supplementary materials for the full list of statements and instructions. Response options ranged from 1 (strongly disagree) to 7 (strongly agree). To assess injunctive norms, we asked participants to rate whether those in their social circles thought that people should ask for consent before engaging in these activities. Once more, scores were calculated for non-BDSM (*α* = 0.82) and BDSM behaviors (*α* = 0.89) separately.

#### Perceptions of Consent Discussions as Disruptive

We asked participants how disruptive they perceived verbal discussions of sexual consent to be by indicating their agreement to six statements (e.g., “I’d find an explicit talk about what someone wants and doesn’t want in a sexual situation really awkward” and “To me, talking about things that are and are not okay in a sexual situation is disruptive”) on a scale from 1 (strongly disagree) to 7 (strongly agree), *α* = 0.78. We developed an original measure of perceptions of consent discussions as disruptive since, to our knowledge, there were no existing measures. We developed the measure by considering the aim and purpose of the measure and consulting with each other iteratively. We then piloted the survey with colleagues and contacts who were part of minority sexual communities, receiving feedback on the clarity and appropriateness of the measure. Based on the feedback received, our measure appeared to be face valid. We assessed convergent validity by inspecting the correlations between our measure of perceived disruptiveness and related consent measures. Perceived disruptiveness was significantly negatively correlated with the likelihood of discussing consent and descriptive and injunctive norms related to discussing consent for BDSM and non-BDSM sexual activities (*r*s < − 0.21), providing further evidence that the measure is valid.

Finally, we conducted a confirmatory factor analysis (CFA) to assess the latent structure of the measure. We conducted a CFA, rather than an exploratory factor analysis, since we developed the model based on previous research as a unidimensional model. As recommended by Sakaluk and Fisher (2019), CFAs are appropriate for assessing the factor structure of theoretically grounded models. The single-factor model provided acceptable fit to the data, TLI = 0.935, CFI = 0.961, SRMR = 0.036, and a RMSEA = 0.081, 90% CI [0.051, 0.113]. Model fit index cut-off values were estimated using the Dynamic Model Fit shiny app (McNeish & Wolf, 2023), which conducts simulations based on our model specifications to estimate appropriate cut-off values. This approach improves upon the default cut-off values provided by Hu and Bentler (1999) which may under or over-estimate model fit as a function of measurement quality.

#### Description of Most Recent Sexual Experience with a New Partner

We asked participants to describe their most recent sexual encounter with a new partner. Participants read the following instructions: “First, we would like you to briefly describe the most recent time you had sex with a new partner. ‘Sex’ can mean many different things so just go with what you personally define as sex. We would like you to describe the most recent time you had sex with a new partner in chronological order and as concretely as possible. Try to use descriptions of concrete behaviors rather than broad terms such as ‘foreplay’ which can mean many different things for different people. When describing the scenario, feel free to include things such as what led up to the sexual activity, whether you talked about sex before it happened and if so, what was said. You could describe what sexual activities took place and what (if anything) was said during sex.” We did not include the term “consent” and asked for “filler” details unrelated to consent to avoid eliciting socially desirable responses (e.g., participants stating that they asked for sexual consent).

#### Sexual Background and Sexual Community Questions

We included two questions measuring sexual experience—participants’ number of sexual partners and how sexually experienced they would say they were on a scale from 1 (not at all experienced) to 7 (very experienced).

Participants then indicated which sexual communities they were part of. We first stated, “There are many sexual groups/communities that you may identify with or be part of. Below we have listed some of the more commonly recognized communities, but this list is certainly not exhaustive. Please tick any of the following you feel you identify with or perceive yourself to be part of, and feel welcome to please write the names of any additional communities that you identify with or are part of.” The options included: BDSM, crossdressing, polyamory, swinger, sex worker, tantra, and other.

Those who indicated that they were part of the BDSM community were additionally asked about their identification with the BDSM community using eight items adapted from Leach et al. (2008) (e.g., “I feel strong ties to other members of the BDSM community,” *α* = 0.71). To measure involvement in the BDSM community, we asked participants how often they participate in BDSM community-related events with others such as “Talk to other members of the BDSM community in person” and “Attend BDSM events (e.g., munches, workshops, play parties)” with the scale-points 0 (never), 1 (once a year or less), 2 (2–6 times a year), 3 (6–12 times a year), 4 (multiple times a month, but less than once a week), 5 (at least once a week but less than daily), and 6 (daily) (*α* = 0.84). In addition, we asked how long (in years and/or months) they had been involved in the BDSM community and how experienced they would rate themselves as a person who engages in BDSM.

#### Demographics

Demographic questions included gender (“What is your gender?”, open-ended), nationality, highest level of education, income (from “far below average “ to “far above average”), political orientation (assessed using three items: “in general,” “regarding economic issues,” and “regarding social issues,” response options ranging from 1 = “Very liberal/left wing” to 11 = “Very conservative/right wing,” *α* = 0.89), religiosity (measured using two items: “Check how often you attend religious worship services” from 1 “less than several times a year” to 5 “more than once a week” and “Tick the number which indicates how important your religion is to you” on a scale from 1 “not at all/have no religion” to 9 “Extremely important; my religious faith is the center of my entire life” *ρ* = 0.79), and relationship status (“Are you currently in one or more committed relationships?”). We also asked participants, “Are you a member of the LGBTIQ+ community?” (Yes/No), and for participants who selected “Yes,” we asked them to select one or more of the following options: lesbian, gay, bisexual, pansexual, asexual, trans*, intersex, queer, non-binary gender identity (e.g., genderqueer, agender, genderfluid), questioning, or other. Finally, participants responded to a question asking where they had heard about the survey.

### Data Analysis Strategy

#### Preliminary Analyses

We categorized participants into three groups: (1) BDSM participants (participants who indicated that they are part of the BDSM community, *n* = 116), (2) other sexual minority participants (participants who indicated that they are not part of the BDSM community but are part of another sexual minority community and/or the LGBTIQ+ community, *n* = 114), and (3) majority participants (participants who did not indicate any membership with the BDSM, fetish/kink, or LGBTIQ+ communities, *n* = 158). Our main analyses focused on comparing these three groups.

We use the term “other sexual minority” to refer to people whose sexual experiences may not align with the traditional, heterosexual sexual script. While some members of the LGBTIQ+ group may not have identified as a sexual minority (e.g., trans participants), we include them in the group “other sexual minority” since their sexual experiences are likely less strongly tied to the traditional heterosexual script, which assumes a gender binary (de Heer et al., 2021).

As we are interested in the comparison of sexual consent norms and behaviors of members of the BDSM community with other groups, it is important to assess whether the different groups differ in other ways and, if so, to control for these differences in our main analyses. We, therefore, ran a series of one-way ANOVAs to test whether sexual communities differed in age, education, income, political orientation, religiosity, sexual experience, and number of sexual partners.^2^ We also ran logistic regression analyses to assess whether there were significant group differences in participants’ gender and relationship status.

#### Coding of Descriptions of Most Recent Sexual Experience with a New Partner

As the number of responses that mentioned BDSM behaviors was small, we focused on non-BDSM behaviors only. Responses that did not provide enough detail (*n* = 103) or did not describe the first encounter with a new partner (*n* = 5). As our focus is on consensual sex, we also excluded responses that included behaviors that violated consent (e.g., partner removed condom against participants’ will, *n* = 2). Lastly, we excluded professional encounters (i.e., sex work encounters, *n* = 3). The final sample size was 275 for the analyses of the descriptions of the most recent sexual experience with a new partner (*n*_BDSM_ = 81, *n*_other-minority_ = 83, *n*_majority_ = 111).

We coded responses using two methods. First, two independent coders coded responses based on whether participants reported explicitly asking for consent. We developed a coding dictionary and procedure that we provided to the two coders. We instructed coders to note instances of verbal consent, which included “a verbal question asked before or during a sexual act relating to whether it is acceptable/enjoyable,” “a verbal statement asking or inviting the partner to start an activity or keep doing it,” or “a verbal statement asking a partner to stop or change an activity.” We provided examples of verbal consent questions, including “Would you like to…?”, “Can I…”, “I want you to…”, and “Is this ok?”, “Do you like this?”

Additionally, we coded a discussion of safe words as a discussion of consent. Coders noted whether participants described asking for consent for sex in general (e.g., “I asked if she was ready to have intercourse, and she said she was”) and/or for specific behaviors (e.g., removing clothes; “I asked her if it was alright to take off her pajama pants”). We instructed coders to focus on whether or not the participant/narrator asked for consent, not whether their sexual partner(s) asked for consent, since sexual partners may be from different sexual communities.

When coders were unsure of a coding procedure or code, they communicated with the first authors. Our resulting measure of consent behaviors is a dichotomous measure of whether consent was discussed (yes/no). Due to the low frequency of consent discussions for specific behaviors, we did not create consent measures for distinct behaviors. After a first round of independent coding, we calculated inter-coder reliability using Cohen’s Kappa. Reliability was above the acceptable threshold of 0.70 (Kappa = 0.76). Coders met to resolve remaining inconsistencies. Finally, a third team member reviewed the codes to ensure they were consistent with the coding dictionary and procedure.

During coding, we noted gray areas when classifying instances of consent. For example, participants described discussing “likes and dislikes.” It is unclear, based on this alone, whether discussions included consent. We, therefore, adopted a second approach to coding consent. In addition to manually coding responses, we used natural language processing to estimate the proportion of each response that included communication-related terms. Each person was assigned a “communication score.” To do this, first, each participant’s text response was converted to a numeric representation using the word embedding model BERT, as implemented in the text package for R (Kjell et al., 2021). Second, using the textSimilarityNorm() function in R, each response was assigned a score based on the semantic similarity of participants’ responses to a collection of communication-related terms, including “ask,” “chat,” “discuss,” and “explain.” This approach allowed us to capture nuances in the extent to which people report communicating during their sexual experience, regardless of whether they explicitly discussed consent. The supplementary materials include details about this procedure, including all communication-related terms used in our model.

#### Main Analyses

In all main analyses, we include relevant control variables.

To test H1a and 1b: “Compared to people who don’t belong to the BDSM community, members of the BDSM community will be more likely to view explicit discussions of consent for non-BDSM behaviors as more descriptively and injunctively normative,” we conducted two 3 (Group: BDSM vs. other minority vs. majority) X 2 (Sexual behaviors: BDSM vs. non-BDSM) repeated measures ANCOVAs, with descriptive norms and injunctive norms as outcome variables, in separate models. To test H1c: “Compared to people who don’t belong to the BDSM community, members of the BDSM community will be more likely to view explicit discussions of consent for non-BDSM behaviors as less disruptive,” we conducted an ANCOVA with group as the predictor and perceived disruptiveness as the outcome.

To test H2: “Compared to people who don’t belong to the BDSM community, members of the BDSM community will be more likely to describe an explicit consent discussion when recounting a recent sexual experience with a new partner,” we first ran logistic regressions, with group (3 levels: BDSM, other sexual minority, and majority; dummy-coded with BDSM as the reference category) as the predictor and consent discussed as the outcome variable (consent discussed/consent not discussed). Second, we ran an ANCOVA, with group as the predictor and communication score as the outcome variable.

#### Sensitivity Analyses

BDSM and other sexual minority participants may have identified with multiple groups (e.g., BDSM and LGBTIQ+). In our sample, 84 of the 116 participants who identified with the BDSM community also identified with another sexual minority community. While our primary research question focused on whether consent norms were different among members of the BDSM community compared to non-BDSM community members, we also tested the independent effects of being a member of the BDSM community, a member of the LGBTIQ+ community, and a member of another sexual minority community (fetish, poly, sex work, or swinger communities). We created dummy-coded variables to indicate group membership and included these variables in regression models predicting our key outcome measures. These models included terms for BDSM community member (1) or not (0), LGBTIQ+ community member (1) or not (0), and other sexual sexuality minority community member (1) or not (0). This analysis allowed us to assess the independent effects of different group memberships, controlling for other forms of group membership. We note discrepancies between these models and those presented in our main analyses. See the online code and analysis file available on the OSF for detailed results https://osf.io/tr4hw/?view_only=d458d3da205d47d0b52d5e4102869b24).

There is conceptual overlap between some control variables (e.g., number of sexual partners) and group membership (e.g., participation in the polyamory community), such that controlling for variables such as number of sexual partners may reduce the variance attributable to group membership. We, therefore, also tested the effects of group membership on consent discussions, including one control variable at a time. These analyses allowed us to test the effects of group membership on consent discussions, isolating the effect of each control variable. We also report the results of simple regressions with no covariates. We report the results of these sensitivity analyses in aggregate below. See the online code and analysis file available on the OSF for detailed results https://osf.io/tr4hw/?view_only=d458d3da205d47d0b52d5e4102869b24).

## Results

### Preliminary Analyses

The groups did not differ in levels of education, income, or sexual experience. The three groups differed in age, *F*(2, 385) = 3.68, *p* = .026, and post hoc tests (LSD) revealed that this was driven by other sexual minority participants who were significantly older (*M* = 34.16, *SE* = 1.24) than both BDSM (*M* = 30.36, *SE* = 1.23), *p* = .076, and majority participants (*M* = 30.03, *SE* = 1.05), *p* = .031. BDSM and majority participants did not differ, *p* = .977.

The groups also differed in political ideology, *F*(2, 348) = 13.49, *p* < .001. Post hoc tests indicated that majority participants (*M* = 4.74, SE = 0.19) were significantly more conservative than both BDSM (*M* = 3.48, SE = 0.22), *p* < .001, and other minority participants (*M* = 3.46, SE = 0.22), *p* < .001.^3^ In contrast, the latter two groups did not differ from each other, *p* = .999. Similarly, the groups differed in religiosity, *F*(2, 340) = 6.13, *p* = .002, such that majority participants (*M* = 0.37, SE = 0.02) were significantly more religious than both BDSM (*M* = 0.29, SE = 0.02), *p* = .015, and other minority participants (*M* = 0.29, SE = 0.02), *p* = .006.^4^ The latter two groups did not differ from each other, *p* = .976.

Lastly, the groups differed in the number of sexual partners they had, *F*(2, 355) = 7.10, *p* = .001. Majority participants (*M* = 10.73, SE = 1.53) reported fewer sexual partners than both BDSM (*M* = 17.69, *SE* = 1.86), *p* = .011, and other minority participants (*M* = 18.79, SE = 1.82), *p* = 0.002. BDSM and other minority participants did not differ, *p* = .906.

Next, we ran logistic regressions to examine whether the three groups differed in gender composition and relationship status. The groups did not differ in gender composition, *p* = .109, or relationship status, *p* = .524.^5^

Based on these results, we controlled for age, political orientation, religiosity, and number of sexual partners in our main analyses. When assessing consent discussions, we also controlled for the word count of participants’ responses to account for the fact that participants who wrote longer responses may be more likely to describe consent and/or sexual communication as a function of writing more words.

Descriptive statistics of our dependent variables, both overall and divided by group, can be found in Table 1. Only 31% of participants described discussing consent at all. Moreover, participants mentioned explicit consent discussions for only 8.49% of coded individual behaviors. The low base rate of explicit consent discussions contrasts with participants’ ratings of such discussions as normative—even for non-BDSM behaviors, both descriptive and injunctive norms were above the midpoint.

**Table 1:** Means and standard deviations for dependent variables

	Overall		BDSM community		Other minority community		Majority	
	*M*	SD	*M*	SD	*M*	SD	*M*	SD
Explicit consent (0 = no, 1 = yes)^a^	31%	0.46	40%	0.49	29%	0.46	25%	0.44
Communication^a^	0.57	0.04	0.57	0.04	0.58	0.04	0.56	0.03
Descriptive norms (non-BDSM)	4.46	1.09	4.60	1.09	4.47	1.07	4.36	1.09
Descriptive norms (BDSM)	5.47	1.18	5.71	1.22	5.38	1.21	5.35	1.09
Injunctive norms (non-BDSM)	4.84	1.13	4.86	1.13	4.78	1.14	4.87	1.12
Injunctive norms (BDSM)	5.92	1.06	5.99	1.16	5.84	1.01	5.93	1.01
Disruptiveness	2.62	1.11	2.37	1.03	2.63	1.10	2.79	1.16

^a^Means and standard deviations for the overall sample were calculated using a smaller sample of eligible responses (*n*s ranging from 267 to 275). Communication scores were continuous. Response options for descriptive norms, injunctive norms, and disruptiveness ranged from 1 (strongly disagree) to 7 (strongly agree)

Table 2 reports bivariate correlations between our dependent variables. Norms and perceived disruptiveness were highly correlated. Explicit consent discussion and communication were significantly positively correlated, such that people who asked for sexual consent tended to have higher scores on communication. Explicit consent discussion was significantly positively associated with injunctive norms (BDSM). People who reported asking for sexual consent also believed that people in their social circles should ask for consent when engaging in BDSM-related sexual activities. Explicit consent was significantly negatively associated with perceived disruptiveness, such that people who reported asking for consent were more likely to view consent discussions as non-disruptive.

**Table 2:** Bivariate correlations

	2	3	4	5	6	7
1. Explicit consent (0 = no, 1 = yes)^a^	.21***	.13*	.09	.12	.13*	− .21***
2. Communication^a^		− .06	− .01	− .12*	− .01	− .02
3. Descriptive norms (non-BDSM)			.55***	.73***	.55***	− .39***
4. Descriptive norms (BDSM)				.40***	.61***	− .27***
5. Injunctive norms (non-BDSM)					.69***	− .29***
6. Injunctive norms (BDSM)						− .25***
7. Disruptiveness						

*p < .05, **p < .01, ***p < .001

^a^Correlations were calculated using data from eligible responses (*n*s ranging from 267 to 275)

### Main Analyses

#### Hypotheses 1a and 1b: Descriptive and Injunctive Norms

For descriptive norms, we found a main effect of sexual behaviors, *F*(1, 300) = 28.70, *p* < .001, *η*_*p*_^2^ = 0.09, such that participants generally believed that people in their social circles were more likely to verbally ask for consent for BDSM activities than for non-BDSM activities. Moreover, in line with our predictions, we also found a main effect of group, *F*(1, 300) = 3.20, *p* = .042, *η*_*p*_^2^ = 0.02. Post hoc tests (Tukey) indicated that BDSM participants generally believed that people in their social circles were more likely to verbally ask for consent compared to majority participants, *p* = .043.^6^ There were no other group differences. The interaction between the group membership and sexual behaviors was not significant, *F*(1, 300) = 2.16, *p* = .117, *η*_*p*_^2^ = 0.01, indicating that the extent to which the groups considered verbal consent discussions to be normative did not vary depending on whether the activities were BDSM or not.

For injunctive norms, results were less supportive of our hypotheses. We once again found a main effect of type of sexual behavior, *F*(1, 294) = 26.84, *p* < .001, *η*_*p*_^2^ = 0.08, such that participants generally believed that people in their social circles were more likely to endorse verbally asking for consent for BDSM behaviors than for non-BDSM behaviors. However, neither the main effect of group, *F*(2, 294) = 0.58, *p* = .550, *η*_*p*_^2^ < 0.01, nor the interaction between group and type of behavior,* F*(2, 394) = 1.24, *p* = .291, *η*_*p*_^2^ < 0.01, were significant. See Main effects and pairwise comparisons are reported in Tables 3 and 4.

**Table 3:** Between-person effects of group membership and covariates on descriptive and injunctive consent norms and perceived disruptiveness of consent discussions

Variable	Descriptive norms			Injunctive norms			Perceived disruptiveness		
	df	*F*	*p*	df	*F*	*p*	df	*F*	*p*
Political ideology	1	2.09	.149	1	6.00	.015	1	0.42	.519
Religiosity	1	0.36	.549	1	0.21	.650	1	0.51	.0476
Age	1	2.17	.142	1	3.73	.054	1	1.24	.266
Number of sexual partners	1	0.87	.352	1	3.66	.057	1	2.79	.098
Group	2	3.20	.042	2	0.58	.560	2	2.20	.038

Group: BDSM, other sexual minority, sexual majority. Results for descriptive and injunctive norms are collapsed across BDSM and non-BDSM sexual behaviors

**Table 4:** Pairwise comparisons between BDSM, sexual minority, and sexual majority participants on descriptive and injunctive consent norms and perceived disruptiveness of consent discussions

Group	Descriptive norms			Injunctive norms			Perceived disruptiveness		
	Mean difference	SE	*p*	Mean difference	SE	*p*	Mean difference	SE	*p*
BDSM—other sexual minority	0.26	0.14	.068	0.15	0.15	.299	− 0.27	0.16	.102
BDSM—majority	0.33	0.14	.015	0.04	0.14	.778	− 0.40	0.16	.012
Other sexual minority—majority	− 0.26	0.14	.596	− 0.11	0.14	.434	− 0.13	0.16	.405

#### Hypothesis 1c: Perceived Disruptiveness

We found a significant effect of group membership on perceived disruptiveness, *F*(2, 301) = 4.03, *p* = .019, *η*_*p*_^2^ = 0.03. Members of the BDSM community found verbal discussions of consent less disruptive than other groups, *p* = 0.031. Other minorities did not differ from either group (*p*s > 0.230). We report main effects and pairwise comparisons in Tables 3 and 4.

#### Hypothesis 2: Consent Discussions

##### Explicit Consent Discussion (yes/no)

To test whether consent is discussed explicitly more often in the BDSM community, we ran a binary logistic regression with the dichotomous explicit consent variable as the outcome and group membership (dummy-coded with the BDSM community as the reference category) as the predictor. Group membership was not associated with the likelihood of discussing consent *p*s > 0.199. We found no differences in the likelihood of discussing consent when comparing BDSM participants to other minority participants, *B* = − 0.52, OR = 0.60, *p* = .195, or when comparing them to majority participants, *B* = − 0.48, OR = 0.62, *p* = .221, df = 197. We also found no differences in the likelihood of discussing consent when comparing other sexual minority and majority participants, *B* = 0.03, OR = 1.09, *p* = .937.

##### Communication Score

Second, we tested whether group membership predicted communication scores. We found a significant main effect of group, *F*(2, 215) = 5.53, *p* = 0.004. We detected a significant omnibus effect, despite similar means, because the standard deviations of the estimates were very small. However, follow-up pairwise comparisons indicated no significant group differences in communication scores (*p*s > 0.065).

##### Sensitivity Analyses

When we modeled group membership using dummy-coded variables, we again found no effect of group membership on likelihood of discussing consent using our dichotomous, coded measure. However, we found that participants from other sexual minority communities (fetish, poly, sex work, or swinger communities) had higher communication scores than other participants (*b* = 0.02, SE = 0.01, df = 198, *p* = 0.002). There was no effect of BDSM group membership (*p* = 0.590) or LGBTIQ+ group membership (*p* = 0.912) on communication.

When we modeled consent discussions as the outcome variable (dichotomous), the pattern of results varied depending on the control variable included in the model. When controlling for the effect of age, religiosity, or word count in separate models, or no covariates, BDSM participants were significantly more likely to discuss consent than majority participants (*p*s < 0.038). However, in models that included political orientation or number of sexual partners, group membership did not predict consent discussions (*p*s > 0.064).

When we modeled communication as the outcome variable, the pattern of results again varied depending on the control variable in the model. When controlling for religiosity, number of partners, or word count in separate models, or including no covariates, other sexual minority participants scored significantly higher on communication than majority participants (*p*s < 0.043). However, when controlling for age or political orientation, we found no significant differences between other sexual minority and majority participants (*p*s > 0.074). We also found no significant differences between BDSM participants and majority participants (*p*s > 0.155) and BDSM and other sexual minority participants (*p*s > 0.674) on communication scores. For detailed results, see the analysis code and output available on OSF (https://osf.io/tr4hw/?view_only=d458d3da205d47d0b52d5e4102869b24).

## Discussion

This study explored variability in sexual consent norms between groups. We compared people who were (1) members of the BDSM community (BDSM participants), (2) members of another sexual minority group (other sexual minority participants), and (3) non-members of a sexual minority group (sexual majority participants). Among BDSM participants, consent discussions were significantly more common in their social circles (descriptively normative) than among majority participants. BDSM participants were also significantly less likely to perceive consent discussions to be disruptive during sex relative to majority participants. We, therefore, find support for Hypotheses 1a and 1c.

Contrary to expectations, we found no significant group differences in the extent to which explicit consent should be discussed (injunctive norms). We also found no group differences in the likelihood of discussing consent when participants described their most recent sexual experience with a new partner; however, there were limitations of our measure of consent discussions, discussed in detail below. We, therefore, did not find support for Hypothesis 1b or 2.

Sensitivity analyses revealed that results varied depending on the control variables included in the models. In three of five follow-up models, BDSM participants were significantly more likely to discuss consent compared to majority participants, and communication scores were significantly higher among other sexual minority participants compared to majority participants. Below, we discuss how these results contribute to existing research on BDSM and consent and possible explanations for the group differences and similarities.

### Consent Discussions Were Considered More Common and Less Disruptive among Members of the BDSM Community

Consistent with previous theorizing and research, we found a small effect of group membership on perceived norms, whereby participants who were members of the BDSM community considered consent discussions as more common, or normative, in their social circles compared to majority participants. This effect did not differ by sexual activity. BDSM participants, therefore, perceived consent discussions during BDSM and non-BDSM activities to be more common in their social circles compared to majority participants. This finding speaks to the potential for identity to shape sexual consent norms and for these norms to extend beyond group-specific activities. The expectation that people will ask for sexual consent appears to spillover from BDSM-related activities to non-BDSM-related sexual activities.

Further, we found a small effect of group membership on perceptions of consent as disruptive. BDSM participants considered sexual consent discussions significantly less disruptive than majority participants. The non-disruptive nature of sexual consent was reflected in participants’ descriptions of their most recent sexual experience with a new partner. For example, one participant from the BDSM community stated, “I asked if she was ready to have intercourse, and she said she was.” This quote describes an explicit, non-disruptive consent discussion. No participant who described a consent discussion described it as disruptive.

For BDSM participants, sexual consent discussions are consistent with their sexual scripts and/or expectations for sexual encounters. However, for majority participants, sexual consent discussions are not a part of the traditional sexual script, and as such, may be perceived as less fluid, less natural, and more disruptive. A related possibility is that BDSM participants have more experience asking for and receiving sexual consent. As such, this familiarity with consent discussions may contribute to perceiving them as normal, expected, and non-disruptive. Interestingly, mean levels of perceived disruptiveness were relatively low across the groups. There are a number of explanations for this. One possibility is that participants were responding in a socially desirable way. Another possibility is that our sample was particularly sexually progressive. Thus, there may be a more market group difference in perceived disruptiveness among larger samples, or samples who are incentivized to respond honestly.

We found no differences between other sexual minority participants and BDSM participants or majority participants. These null findings may be related to the heterogeneity of this group. There is a large amount of variability within the group of other sexual minority. As such, there is also likely a large degree of variability in consent discussions.

### No Group Differences in the Likelihood of Describing Consent Discussions

We hypothesized that participants in the BDSM community would be more likely to report explicit consent discussions relative to other groups because BDSM activities are defined by the inclusion of consent (Carlström, 2017). We may not have found support for Hypothesis 2 because we used an indirect measure to assess consent discussions. We asked participants to describe their most recent sexual experience with a new partner in detail, including “whether you talked about sex before it happened and, if so, what was said,” and “what (if anything) was said during sex.” However, we did not directly ask whether they explicitly discussed consent to limit socially desirable responses. Some participants may have simply left this information out. Thus, this measure may be a better indicator of the salience of consent when describing a recent sexual encounter. Another possibility is that participants could not clearly remember whether or not they discussed consent, as one participant noted. As such, there may have been group differences in the actual rates of consent discussions that were not reflected in the re-tellings by participants.

In addition to participants potentially omitting information, we were limited in our ability to code responses. We focused on whether participants asked for sexual consent, not their partner(s) because it is unclear what group their sexual partners belonged to. As such, we did not include instances of being asked for consent among the BDSM community in our qualitative analyses. However, we were able to capture the extent to which both partners communicated via natural language processing.

Another possibility is that we recruited a sexually progressive sample. Participants who agreed to participate in a study on sexual attitudes and behaviors may have more sexual experience, be more comfortable discussing sex, and may be a part of social circles that are sexually progressive. These factors may have increased the likelihood that our participants in all groups would be more likely to discuss consent or communicate about sex more generally. However, the proportion of people who discussed consent was relatively low, suggesting that our analyses were likely not constrained by ceiling effects. Interestingly, when we did not include political orientation as a covariate, BDSM participants were more likely to discuss consent, and other sexual minority participants had higher communication scores compared to majority participants. Group differences in consent norms may be driven by the extent to which participants are sexually liberal, regardless of other sexual community identities.

Finally, we note that we coded very few instances of BDSM activities since we were interested in assessing consent norms across both BDSM and non-BDSM sexual experiences. Thus, explicit consent may have been either less common or felt less important to report when describing the sexual encounter. Our findings regarding group differences in consent discussions, therefore, reflect an indirect, preliminary measure of consent, and additional research is needed to further test for group differences in the frequency of consent discussions.

### No Group Differences in Injunctive Norms Toward Consent

Another unexpected finding was that we found no group differences in the extent to which consent should be discussed among participants’ social circles. It is promising that, in general, mean scores were above the scale's mid-point, suggesting that participants felt that sexual consent should be a feature of others’ sexual experiences. While the mean was relatively high, it was not at the upper limit of the scale, suggesting that our findings were likely not constrained by ceiling effects. There may be a consensus across groups that sexual consent should be obtained during sex. These findings suggest that efforts to shift sexual consent norms should not necessarily focus on convincing people that sexual consent is important. Rather, it may be more impactful to shift identities and descriptive norms, as well as education on how to engage in consent, to increase the frequency of consent discussions. If a primary source of sex education for young people is media depictions of sex, one potentially impactful change would be to include consent discussions in portrayals of sexual experiences. Another avenue for change may be formal sex education that emphasizes community norms and expectations of discussing consent.

### Implications

Our findings support a social identity approach to understanding sexual consent norms (Ellemers & Haslam, 2012; Terry et al., 1999). Group membership is associated with differences in the likelihood of perceiving sexual consent as descriptively normative and disruptive. One possibility is that people in the BDSM community practice consent in the context of BDSM sex only because of a sexual script that is specific to that context. However, identifying with the BDSM community is associated with perceptions of consent as more common for BDSM and non-BDSM sexual activities. This spillover, or generalized effect, may speak to the importance of identity and experience in shaping behaviors. People who identify with the BDSM community may be more likely to perceive consent as a key component of sex because of the norms and practices within their group.

Our findings also speak to the variability, and malleability, of consent norms. Consent discussions are, of course, not inherently disruptive and are considered a natural part of sexual encounters for many people. Changing how sex is depicted and discussed is one way consent norms may be shifted among majority people. The popularity of the 50 Shades of Grey novels and films speaks to an interest in BDSM sex. One promising avenue for change is to include explicit consent discussions in such representations of BDSM sex (and non-BDSM sex) in the media. Such exposure may serve as an indirect education on discussing sexual consent. Engaging in consent psychoeducation may be particularly important if people’s interests in fictional BDSM accounts then extend to engaging in BDSM-related sexual activities. If people engage in BDSM-related sexual activities but do not identify as someone who engages in BDSM, they may miss the crucial component of sexual consent. Further, popular depictions of sexual consent may also provide indirect education and modeling for people who identify with the BDSM community, as consent isn’t always obtained within the BDSM community (Dunkley & Brotto, 2020).

Finally, we note that sexual consent discussions are likely relevant for addressing the high rates of sexual misconduct and abuse. When consent is perceived to be descriptively and injunctively normative, and people are engaging in sexual consent discussions, instances of incorrectly “assumed consent” are less likely. Further, sexual experiences may be more likely to focus on mutual pleasure when likes and dislikes are openly discussed and saying yes and no to certain acts becomes more fluid. However, changing consent norms should not be the only focus in the efforts to address issues of non-consensual sex. Indeed, when sexual experiences are non-consensual, the victim may communicate this clearly. Efforts to address the likelihood that someone will perpetrate sexual violence or harassment should complement consent education (Rozee & Koss, 2001). Such efforts are complex and require addressing worldviews often rooted in misogyny and entitlement (Abbey et al., 2011; Gavey, 2018; MacKinnon, 2018).

### Limitations and Future Directions

Our study examined sexual consent using indirect measures. Our results are, therefore, limited in their ability to determine how the frequency of consent discussions differs between groups. One possibility for future research is to conduct in-depth interviews with sexually diverse populations. Interviews would allow participants to describe when consent is discussed (if at all), who asks for sexual consent, and what sexual consent means to them. The interviewer could also clarify what participants mean when they describe talking about “likes and dislikes.” Further, such a study could provide insights into the sexual communities of participants’ partners and how group identity might shape consent discussions across partners.

Another possibility is to quantitatively measure the frequency of asking for sexual consent. It may be possible to assess social desirability biases by prompting participants to provide answers that are as honest and accurate as possible and reminding them that their answers are completely anonymous. Future research could ask participants how long ago their sexual encounter was to account for inaccuracies due to memory.

We assessed descriptive and injunctive norms by asking participants to consider how people in their social circles would and should act. Participants who identified strongly with the BDSM community likely included other members of the BDSM community in the conceptualization of their social circle. However, the question did not specify which social circles participants should think about, and some participants may have imagined broad or specific social circles that may not have represented their reported sexual community. Future research may reduce potential error variance by asking about the norms of specific sexual communities.

Our findings regarding the perception of sexual consent as important and something that people should be engaging with provides hope for updating sexual scripts. One avenue for future research is to explore ways to increase a person’s likelihood of engaging in consent discussions during sex. For example, it may be possible to make salient an identity that incorporates sexual consent discussions. Asking participants whether they identify as feminists or ethical actors in sexual scenarios may promote willingness to engage in sexual consent discussions. Future work could also assess the effect of viewing sexual consent discussions in media depictions of sex.

### Conclusion

Sexual consent is essential to engaging in ethical, pleasurable sex for all parties. However, sexual consent discussions are not integrated into the traditional sexual script and, typically, haven’t been depicted on screen or in popular fiction. This study explored whether sexual consent may be more common among members of the BDSM community who engage in non-traditional sex that is defined, in part, by consent. We found that, regardless of whether sex included BDSM activities, members of the BDSM community considered consent discussions more normative than majority participants. Interestingly, there were no group differences in the extent to which people thought consent should be discussed. Our findings suggest that people perceive consent discussions to be important and that there is a need for the traditional sexual script to be updated to incorporate consent discussions.

### Supplementary Information

Below is the link to the electronic supplementary material.

Supplementary file1 (DOCX 18 kb)

## Data Availability

Available upon request.

## Footnotes

1 We hypothesized potential mechanisms for group differences in consent discussions, which we note in the Online Supplementary Materials.

2 Sex workers were excluded from group analyses comparing number of sexual partners and sexual experience.

3 This was measured on an 11-point scale with higher numbers corresponding to more conservative views.

4 Religiosity is given on a scale from 0–1 with higher numbers indicating stronger religiosity.

5 Non-binary participants were excluded from this analyses due to small sample size.

6 Post hoc tests did not include covariates.

## Code Availability

Code and output are available on the study OSF page, https://osf.io/tr4hw/?view_only=d458d3da205d47d0b52d5e4102869b24
