# Supplementary material for: Sexual Consent Norms in a Sexually Diverse Sample
Source: Arch Sex Behav. 2023 Nov 28;53(2):577–92. doi: 10.1007/s10508-023-02741-0 (PMC10844416; doi:10.1007/s10508-023-02741-0)
Supplement: Supplementary file 1 — Supplementary file1 (DOCX 18 kb) [file 10508_2023_2741_MOESM1_ESM.docx]

Online Supplementary Materials

Hypotheses

In addition to H1 and H2 reported in the manuscript, we developed a third hypothesis:

H3: Norms and disruptiveness mediate the effect of BDSM community membership on the likelihood that sexual consent is explicitly discussed.

Since we did not find group differences in the likelihood of discussing consent in our study, we did not test for mediation.

Method

Measures

Description of Most Recent Sexual Event. Participants read the following instructions: “read the following instructions: “First, we would like you to briefly describe the most recent time you had sex with a new partner. “Sex” can mean many different things so just go with what you personally define as sex. We would like you to describe the most recent time you had sex with a new partner in chronological order and as concretely as possible. Try to use descriptions of concrete behaviors rather than broad terms such as “foreplay” which can mean many different things for different people. When describing the scenario, feel free to include things such as what led up to the sexual activity, whether you talked about sex before it happened and if so, what was said. You could describe what sexual activities took place and what (if anything) was said during sex. This task should not take longer than 10 minutes. Feel free to stop after that time even if you could still think of more details. It is of course also fine if you take much shorter or longer than 10 minutes – this is merely a guide.”

**Descriptive Norms.** Participants were presented with the following instructions: “The following questions will ask about how people in your social circles feel and act when it comes to having sex with a new partner. If you are a member of many different social circles, think of the social circle you identify with the most. We know that it can be hard to know what other people might be doing or thinking, but just try to guess if you’re not sure. There are no right or wrong answers to these questions. Some of the questions will relate to communication during sex. In the following questions, we want to focus mostly on verbal communication. If you are unsure how to interpret a word like “ask”, “discuss”, “let someone know” etc., please interpret this as asking/discussing/letting know verbally. Please read each statement carefully before rating your agreement on a scale ranging from 1 (strongly disagree) to 7 (strongly agree). First, we are interested in how you think most people in your social circles would behave when having sex with a new partner. Please focus on how people would act, even if they don’t actually engage in these behaviors.” The list of statements provided were preceded with “Most people in my social circles…” See Table S1 for a full list of statements.

**Injunctive norms.** We included the same list of items as those used to assess descriptive norms, however, instead of using the word ‘would’, we reframed the questions to reflect whether people *should* ask for consent. For example, we asked participants to rate their agreement with the statements, “Most people in my social circles…” “…think that people should ask a new partner if they would like to have sex even if they seem turned on during foreplay”, and “…...think that it’s necessary to ask a new partner if they are ok with mild biting during sex.” Response options ranged from 1 (strongly disagree) to 7 (strongly agree).

**Perceptions of Consent Discussions as Disruptive.** We asked participants to indicate their agreement with the following six statements: “I’d find an explicit talk about what someone wants and doesn’t want in a sexual situation really awkward”, “I’d feel embarrassed if I explicitly asked a partner for permission in a sexual situation”, “If a partner told me to stop some sexual behavior and asked me to do something else instead, I’d be very turned off”, “I feel comfortable explicitly talking about things I want and don’t want in sexual situations” (reverse coded), “To me, talking about things that are and are not okay in a sexual situation is disruptive”, and “I feel comfortable asking my sexual partner for permission in sexual situations” (reverse coded). Response options ranged from 1 (strongly disagree) to 7 (strongly agree).

**Natural Language Processing**

Each participant's text response was converted to a numeric representation using the word embedding model BERT, as implemented in the *text* package for R (Kjell et al., 2021). Using the *textSimilarityNorm()* function, We then computed the semantic similarity between the vector representation of each participant's response, and a vector representing a set of communication related-words to estimate the prevalence of communication-related content.

We assessed “communication” using the following terms:
"answer", "answered", "answering", "answers", "ask", "asked", "asking", "chat", "chatted", "chattered", "communicate", "communicated", "communicating", "communication", "communicative", "conversation", "conversations", "discuss", "discussed", "discussing", "discussion", "discussions", "explain", "explained", "explaining", "informed", "listen", "listened", "listening", “mention”, “mentioning”, “mentioned”, "question",  "questions", "said", "say", "saying", "speak", "speaking", "spoke", “stated”, “state”, “stating”, "talk", "talked", "talking", "tell", "telling", "told", "understand", "understood"

References

Kjell, O., Giorgi, S., & Schwartz, H. A. (2021). Text: An R-package for Analyzing and Visualizing Human Language Using Natural Language Processing and Deep Learning. *PsyArXiv Preprint*. https://doi.org/10.31234/osf.io/293kt

| **Table 1**  *Descriptive Norm Items* | |
| --- | --- |
| Non-BDSM activities | BDSM activities |
| ...would ask a new partner if they would like to have sex if they seem turned-on during foreplay  ...would assume that a new partner wants to have sex unless the partner verbally objects*  ..would give a new partner oral sex without asking if they would like it or not*  ...would ask for permission before stimulating a new partner anally  …would ask a new partner what kinds of sexual positions (e.g., doggy style, cowgirl) they are ok with before trying them  ...would rely solely on non-verbal signals from a new partner to judge whether a sexual act is ok or not*  …would talk about what kinds of sexual activities a new partner is comfortable with and not comfortable with before having sex  ...would ask a new partner if they are ok with mild biting during sex  ...would ask a new partner before they hold their wrists down during sex | ...would verbally ask for permission before spanking a new partner  ...would usually ask for permission before tying a new partner up  ...would usually ask things such as “do you want this?” before engaging in behaviors that leave visible marks on a new partner’s body (e.g., bruises or scratches)  ...would ask a new partner if they are ok with being gagged  ...would ask a new partner if they are ok with being blind-folded  ...would ask a new partner if they are ok with receiving commands in sexual situations  ...would ask a new partner if they agree to have certain objects used on them that may inflict pain (e.g. nipple clamps, paddles, hot wax)  ...would ask a new partner if they agree to being physically forced into particular sexual positions (e.g., doggy style, cowgirl) |
| *Note*. Statements followed the words “Most people in my social circles…”. Items marked with an * were reverse-coded | |
